# Outcomes in antiretroviral-naive HIV-infected patients initiating therapy with TDF/FTC plus either atazanavir/r or another third recommended drug

**DOI:** 10.1186/1758-2652-13-S4-P9

**Published:** 2010-11-08

**Authors:** E Billaud, JM Lacombe, A Abgrall, J Ghosn, O Launay, JM Livrozet, JL Meynard, J Pavie, D Costagliola

**Affiliations:** 1 CHU Nantes, Nantes, France; 2 INSERM and Universite Pierre et Marie Curie, U943, Paris, France; 3 AP-HP, CHU Bicetre, Le Kremlin-Bicêtre, France; 4 AP-HP, CHU Cochin, Paris, France;; 5 HCL, CHU Lyon, Lyon, France; 6 AP-HP, CHU Saint-Antoine, Paris, France; 7 AP-HP, CHU Saint-Louis, Paris, France

## Background

Atazanavir/r (ATV/r) is a recommended PI option for treating ART-naive patients in recent guidelines. TDF/FTC is the most commonly used recommended backbone in this setting. Our objectives were to compare times 1) to discontinuation of the third drug, 2) to virologic suppression (VL< 50 copies/ml), with discontinuation of the third drug considered as failure, 3) to an increase of at least 100 CD4 cells/mm^3^, with discontinuation of the third drug considered as failure, 4) to AIDS or death from any cause, with an intention to continue approach 5) to hospitalization, AIDS or death with an intention to continue approach in patients initiating with ATV/r or another recommended third drug plus TDF/FTC.

## Methods

ART-naive patients in the FHDH ANRS CO4 cohort who started cART containing TDF/FTC plus either ATV/r or another recommended third drug (LPV/r, f-AMP/r or EFV) after 31/12/2003 and at least 12 month before the closing date of the database were analyzed. Multivariable Cox's proportional hazards models were used to control for the following potential confounders: Age, sex, geographical origin, transmission group, baseline CD4 cell counts and viral load, AIDS stage, HCV co-infection and year of starting.

## Results

2910 patients (ATV/r=517 and Other=2393) were analyzed, with a median follow-up of 19.1 months (IQR: 9.8-30.4). The third drug was EFV in 1129 (47.2%), LPV/r in 1045 (43.7%) and f-APV/r in 219 (9.2%). Baseline median CD4 was 246 for ATV/r and 228 for other drugs (p=0.0064) and viral load 4.87 for ATV/r and 4.89 for other drugs (p=0.0688). At 24 months, the rates of the different outcomes were for ATV/r versus other drugs:

• Discontinuation of the 3rd drug: 24% and 27% (aHR: 0.76 (0.61-0.94))

• Virologic suppression (VL< 50 copies/ml): 83% and 84% (aHR: 0.93 (0.84-1.04))

• Increase of at least 100 CD4 cells/mm^3^: 83% and 80% (aHR: 1.09 (0.98-1.21))

• AIDS or death: 7% and 10% (aHR: 0.88 (0.61-1.28))

• Hospitalization, AIDS or death: 17% and 21% (aHR: 0.93 (0.73-1.18))

Sensitivity analyses using propensity scores and subgroup analyses in patients with CD4<200/mm^3^ depending of the level of viral load (< or >=100 000 copies/ml) will also be presented.

## Conclusions

In this observational settings in ART-naive patients, use of ATV/r as third drug was associated with a lower rate of discontinuation and with no difference in terms of virologic suppression, immunological and clinical outcomes as compared to other recommended third drugs.

